# Direct coupling: a possible strategy to control fruit production in alternate bearing

**DOI:** 10.1038/srep39890

**Published:** 2017-01-04

**Authors:** Awadhesh Prasad, Kenshi Sakai, Yoshinobu Hoshino

**Affiliations:** 1 Department of Physics and Astrophysics, University of Delhi, Delhi 110007, India; 2 Department of Environmental and Agricultural Engineering, Tokyo University of Agriculture and Technology, Tokyo 183-8509, Japan; 3 Department of Environment Conservation, Tokyo University of Agriculture and Technology, Tokyo 183-8509, Japan

## Abstract

We investigated the theoretical possibility of applying phenomenon of synchronization of coupled nonlinear oscillators to control alternate bearing in citrus. The alternate bearing of fruit crops is a phenomenon in which a year of heavy yield is followed by an extremely light one. This phenomenon has been modeled previously by the resource budget model, which describes a typical nonlinear oscillator of the tent map type. We have demonstrated how direct coupling, which could be practically realized through grafting, contributes to the nonlinear dynamics of alternate bearing, especially phase synchronization. Our results show enhancement of out-of-phase synchronization in production, which depends on initial conditions obtained under the given system parameters. Based on these numerical experiments, we propose a new method to control alternate bearing, say in citrus, thereby enabling stable fruit production. The feasibility of validating the current results through field experimentation is also discussed.

Alternate bearing (biennial bearing) is a common phenomenon in many tree crops, in which a year of heavy crop yield, known as the “on-year” is followed by an “off-year” of extremely light or no yield^1^,^2^. This pattern continues in subsequent years. Synchronization of fruit production observed in orchards and national markets is also called alternate bearing. Alternate bearing in individual trees and orchards is a challenge for farmers because they need advanced skills to maintain constant production^3^. Farmers and agronomists have recognized alternate bearing of tree crops as a complex phenomenon, and various efforts have been made to understand its behavior and mechanisms^4^.

Similar to alternate bearing, the phenomenon of acorn masting has been a common subject in studies of forest and ecological management^5^,^6^,^7^,^8^,^9^. Isagi^10^ was the first to develop a simple nonlinear dynamics model to explain the annual oscillations of acorn production in an individual tree. This model is called the resource budget model (RBM) because it is based on the energy (photosynthate) stored in a plant. Isagi also attempted to explain the synchronization of acorn production in a large population (i.e. masting) by globally coupled RBM oscillators. This coupling is mainly caused by pollination in oak trees.

The RBM model is based on the amount of photosynthate produced each year in an individual plant. The majority of photosynthate is used for the plant’s growth and maintenance. The remaining photosynthate (*P*s) is stored in the plant. The accumulated photosynthate stored in a plant is expressed as *I*. In a year when accumulated photosynthate exceeds a certain threshold *L*_T_, then the remaining amount, *I* – *L*_T_, is used to represent the cost of flowering, *C*_f_. These flowers are then pollinated and bear fruit, the cost of which is designated as *C*_a_. Usually, the fruiting cost is a function of the cost of flowering, i.e., *C*_a_ = *F*(*C*_f_) where *F*(.) is a function. The accumulated photosynthate becomes *I* = *L*_T_ after flowering. Once fruiting is over, the remaining photosynthate becomes *L*_T_−*C*_a_ i.e., *I* = *L*_T_ − *F*(*C*_f_). In the RBM, we consider the function *F*(*C*_f_) to be linear, i.e., *C*_a_ = *R C*_f_ where *R* is a proportionality constant that represents the flowering to fruiting ratio. In this case, the phenomenon is modeled as^10^,^11^,^12^,^13^,^14^,^15^.


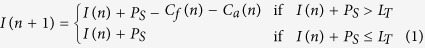



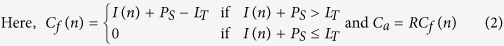


where n = 1, 2, 3…. represents the years. Here *R* is considered as a control parameter for this model. Figure 1 shows a bifurcation diagram, *I*, as a function of the parameter *R*. At lower values of *R* < 1 (in region A1) there is only a single stable solution (Period-1 regime:A1, shown by the solid red line), indicating constant yields across years (i.e., no alternate bearing occurs). As *R* exceeds 1, the fixed point solution becomes unstable. Here, the upper and lower chaotic bands, which individually have an infinite number of bands^16^, correspond to high and low yields respectively (Multi-band chaotic regime:A2).

The RBM has been employed to explain the alternate bearing of *Citrus unshiu*, which is a parthenocarpicy species. Sakai^17^ validated a one-year forward prediction using and an experimental dataset and also applied the nonlinear control theory^18^ to estimate the control input needed to suppress alternate bearing^19^. Recently, some of the dynamical properties of this phenomenon have been uncovered using this model^16^. Attempts to let RBM fit the real behavior of *C. unshiu* have been carried out using experimental data^20^.

The conventional method of producing *C. unshiu* fruit aims to stabilize output by fruit thinning and pruning. In terms of nonlinear dynamics, this can be thought of as stabilizing the state (i.e., fruit production volume) at an unstable fixed point. Therefore, we can think of the conventional production method as equivalent to controlling chaos, similar to the OGY method^18^; though farmers and agronomists surely carry out these procedures based on empirical observations rather than the underlying theory. However, this conventional method still requires farmers to have very advanced skills to be able to determine the appropriate amounts of fruits and/or flowers to remove^3^.

The main objective of this paper is to propose a new fruits production method to get constant total fruits production form an orchard of *Citrus unshiui* by enhancing alternate bearing of individual citrus trees, based on the principles of phase synchronization of coupled oscillators and utilizing the existing RBM^10^,^12^,^13^,^14^,^21^. We propose a production method using direct coupling between citrus trees. In practice, two is the appropriate number of connected trees. Our method (the direct coupling method) aims to realize artificial alternate bearing by grafting two (or more) trees. In a given orchard with N trees, this method incorporates the fact that N/2 trees are experiencing an on-year and the rest an off-year at any given time, which allows the total production of N trees to be constant every year^22^.

We have organized this paper as follows: Section II discusses the interactions between plants under different types of coupling. Section III offers the results for two and three coupled plants. Section IV highlights the feasibility of experimentation. Section V summarizes the results.

## Modeling Plant Interactions

In a real-world plant population, many types of interactions occur among plants, resulting in various types of dynamic behaviors^23^,^24^. Synchronization is one such phenomenon that occurs as a result of interactions between systems. Synchronization happens when plants’ actions are in unison i.e., when their subsystems follow the same life cycle schedule^25^,^26^. This behavior has been well studied and has been found to be present in almost all branches of science^13^,^26^,^27^, including in ecology^28^,^29^,^30^,^31^,^32^. Many types of synchronization, such as complete, lag, intermittent, and generalized synchronization^27^, have been observed as a result of interactions between systems. These have been discussed in detail in the literature^27^. Phase synchronization^27^,^33^ is one such type of synchronization, in which the amplitudes of individual oscillators (e.g. plants) are uncorrelated, while their phase-difference remains bounded. If the phase-difference of the oscillators is near zero, then it is termed “in-phase” behavior. If the phase-difference is near π, then it is called “out-of-phase” behavior^26^,^33^,^34^,^35^,^36^,^37^. In this work, we also explore the possibility of in- and out-of-phase behaviors among plants in the presence of different types of interactions. The models for three types of interactions, indirect, direct and mixed coupling, are discussed below (Fig. 2(a) and (b)). Here, the main target plant is a *Citrus* species. As *Citrus unshiu* is parthenocarpic, the effect of pollen coupling is much smaller than in cross-pollinating species, such as *Citrus hassaku*. Therefore, we took *C. unshiu* as the target for our direct coupling model and *C. hassaku* for the indirect coupling model. If a *C. unshui* orchard were located near to a *C. hassaku* orchard, the *C. unshui* would cross-pollinate and produce seeded fruits from the *C. hassaku* pollen. In other words, the indirect couplings shown in Fig. 2(a) and (b) model the effect of cross-pollination of *Citrus hassaku*.

## Direct interaction (diffusive coupling): grafting

Grafting is an important technique by which plant parts (e.g. branches) are taken from one or more different plants and joined to another plant, the rootstock. Although grafting is a very popular technique, to our knowledge, it has not been used thus far in alternate-bearing plants to control the production. Recently, the joint tree training technique^37^,^38^ was developed to decrease the labor needed for tree training (e.g. brunching, thinning and pruning) of deciduous broad-leaved fruit trees such as pear, peach and apple. The joint tree training technique involves connecting trees along a line by grafting neighboring trees together. As the alternate-bearing in deciduous broad-leaved fruit trees is weaker than in *Citrus* spp.(evergreen trees), the joint training technique does not aim to control alternate bearing of them. Trees can also exhibit natural connections through branches, trunks, and roots^39^,^40^. Figure 2(c) and (d) show representative connections between branches/trunks and roots for different trees, respectively. These are typical examples of the natural physiological integration of plants; this type of interaction is referred to as “direct coupling” in this paper. In this work, we place emphasis on this type of integration and try to understand alternate-bearing behaviors in the presence of direct coupling. Direct coupling should realize physiological integration so that diffusive energy transfer can occur between the coupled plants. Hence, we can describe the direct coupling model as diffusive coupled nonlinear oscillators in eq. (3).


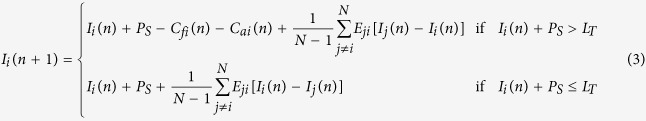


Here, 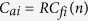,where *E*_ji_ is the coupling strength for the direct interaction between the j^th^ and i^th^ plants. We consider that *E*_ji_ = *ε* in Eq. (3) for simplicity. This coupling is illustrated as solid black arrows in Fig. 2(a,b) between different plants (circles).

## Indirect interaction (mean-filed coupling): pollination

Plants interact with each other via external agents that can be biotic (e.g., animals, birds, fungi or bacteria) or abiotic (e.g., water, light, wind, soil, humidity or gases). Indirect coupling has been well studied in ecology and has been shown to affect plant populations in many ways. For example, pollen interactions with wind and insects enhance seed and/or fruit production in plants^5^,^6^,^7^,^13^,^41^,^42^,^43^,^44^.

The indirect coupled RBM model is expressed


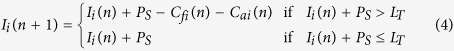


Here, 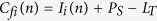 and 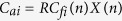 (cf. Eq. (2)). where


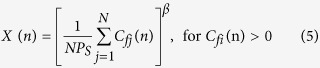



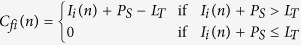


## Mixed interaction: direct and indirect

In this paper, we consider two types of mixed interactions as illustrated in Fig. 2(a) and (b). Figure 2(a) is the interaction of two trees coupled directly and indirectly. Figure 2(b) is a network of three oscillators (plans), in which two plants have direct interaction in addition to the indirect interaction among all three plants. This can be demonstrated by combining Eqs (3) and (5). The mixed interaction models the case in which the orchard of *Citrus unishiu* is located near to a *C. hassaku* orchard.


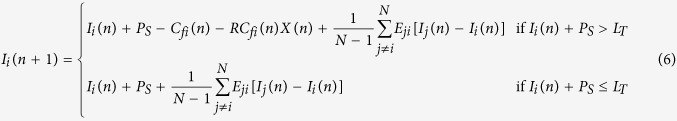


## Results and Discussion

### Effects of direct and indirect interactions in two coupled plants

To show the effect of both types of coupling we first consider two coupled plants (Fig. 2(a)) in the presence of either direct or indirect interactions in the period-1 regime (A1) and multi-band chaotic regime (A2), as alternate bearing is supposed to occur mostly in these two regimes according to experimentally estimated *R* values^11^.

### Period-1 regime, A1: R = 0.5

**Figure 1:**
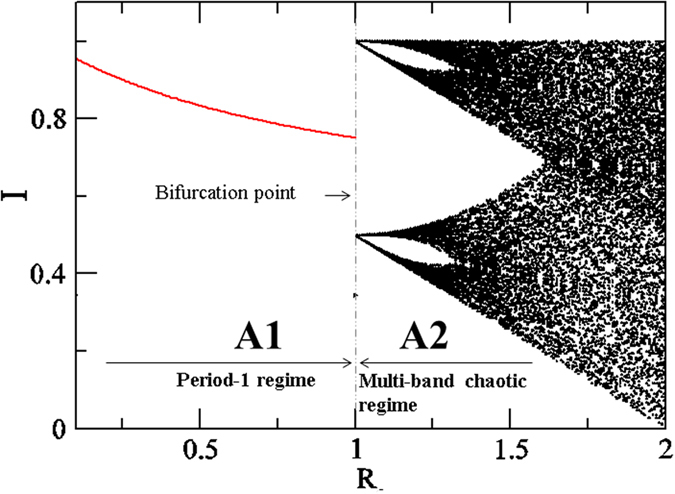
Bifurcation diagram, *I*, showing a function of parameter *R*, for the single-resource budget model,Eq. (1). Regions A1 and A2 correspond to period-1 regime and multi-band chaotic regime, respectively^16^.

To demonstrate the function of direct interaction (coupling), we first consider the period-1 regime *R* < 1 (marked as A1 in Fig. 1) because the conventional RBM does not generate any oscillation in the range of *R* < 1. Note that *R* = 1 is the only clear bifurcation point of the conventional RBM.

Period-2 bifurcates from period-1 at *ε* = 0.25 as shown in Fig. 3(b). This is a characteristic property of directly coupling two RBMs (single oscillators). In region P2, *ε* ≥ 0.25, we find out-of-phase behavior, as shown in Fig. 3(c) at *ε* = 0.4. In A1 (*R* < 1), this out-of-phase behavior occurs for any initial conditions. Figure 3(d) shows the normalized fractions of the initial conditions for either in-phase (*f*_in_) or out-of-phase (*f*_out_). These fractions are calculated as follows. If 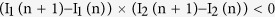, then both plants show out-of-phase behavior; otherwise they display in-phase behavior. We considered 1000 random initial conditions in I_1_∈[0, 1] and I_2_∈[0, 1], and we determined whether they would result in in-phase behavior (*f*_in_) or out-of-phase behavior (*f*_out_). Figure 3(d) clearly shows that *f*_in_ is zero while *f*_out_ is 1.0, which indicates that the plants produce fruits in an out-of-phase manner. This suggests that, with direct coupling, plants can be switched to an out-of-phase mode of fruit production even under Period-1 regime (A1) shown in Fig. 1.

### Multi-band chaotic regime, A2: *R* = 1.2

To show the dynamics of two coupled plants when they are in a multi-band chaotic regime (A2) in Fig. 1, we considered that *R* = 1.2. This value is very close to that estimated using experimental data for *C. unshiu* yield^11^. At this parameter value (*R* = 1.2), there are many chaotic bands; however these are separated in two parts: above and below the unstable period-1 fixed point^16^. At *R* = 1.2, individual plants show alternate bearing that appears cyclic (i.e., with “on-years” followed by“off-years”). However, the precise fruit-bearing behaviors are actually chaotic. The results for coupled plants when *R* = 1.2 are shown in Figs 4 and 5 for direct and indirect couplings, respectively.

#### Direct coupling RBM

Here, we discuss the dynamic behavior of direct coupling RBMs. The results of direct coupling are shown in Fig. 4. The bifurcation diagram, Fig. 4(a), shows that multi-band chaos starts merging and becomes single-band chaos at approximately *ε* = 0.25. The fractions of the initial conditions (Fig. 4(b)) show that there is always an increase in out-of-phase motion as coupling strength *ε* is increased (i.e. direct coupling induces out-of-phase motion at higher coupling strengths *ε*). However, within this given parameter range, both in- and out-of-phase behaviors coexist. Figure 4(c) shows the basins of these coexisting attractors, in- and out-of-phase, in black and red, respectively, at *ε* = 0.1. The typical trajectories of in-and out-of-phase behaviors are shown in Fig. 4(d) and (e), respectively, at *ε* = 0.1. For the alternate bearing of *C. unshiu*, we hypothesize that pollen coupling will have little effect because the species is parthenocarpic. Therefore, the results obtained here are very useful for developing an improved method for producing *C. unshiu* fruits. In conventional citrus production, citrus trees are never joined by grafting or other methods. Therefore, the ratio of in-phase to out-of-phase trees is 1:1 as we can see in Fig. 4(b) at *ε* = 0. The strength of direct coupling *ε* should be estimated in field experiments. Physiological integration is expected when grafting two citrus trees. Thus, we expect to realize a positive value of *ε* in field experiments.

#### Indirect coupling RBM

Figure 5(a) shows the bifurcation diagram of *I*_1_ with the control parameter *β*, which demonstrates that multi-band chaos continues to exist; however, periodic windows occur in between. Figure 5(b) shows the fractions of out-of-phase and in-phase states with *f*_out_ and *f*_in_. Note that owing to indirect interaction (pollen), we only obtain an in-phase solution after *β* = 0.25, which indicates that indirect coupling induces in-phase behavior at higher coupling strengths. Below *β* = 0.25, we obtain coexisting attractors. Figure 5(c) shows the basins of these coexisting attractors at *β* = 0.1, where the black and red regions show the initial conditions leading to the in-phase and out-of-phase states, respectively. Figure 5(d) and (e) show the typical trajectories for coexisting in-phase and out-of-phase attractors, respectively, at *β* = 0.1.

### Effect of mixed interactions in two plants

**Figure 2:**
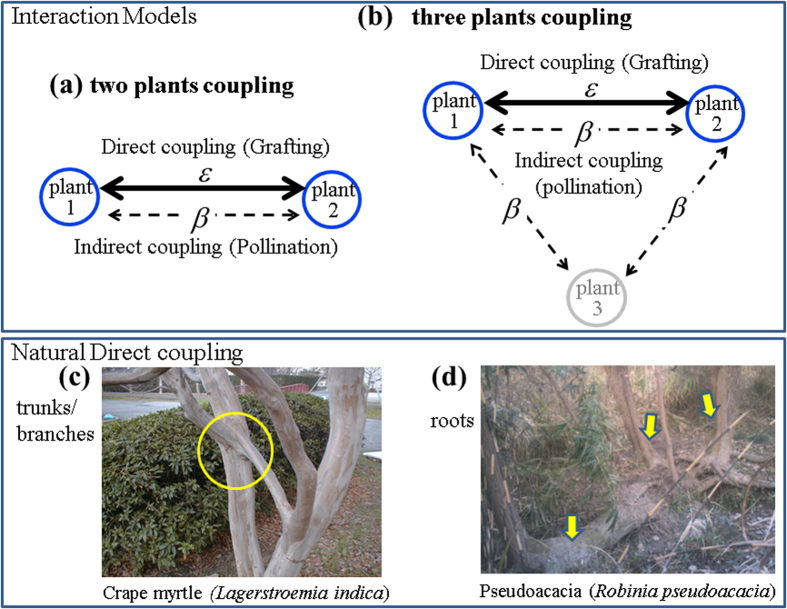
Schematic drawing and real images for interactions. (**a**) Two plants and (**b**) three plants. The solid and dashed arrows indicate direct and indirect interactions, respectively. The representative photographs show direct coupling between (**c**) the trunks/branches of Crape myrtle (*Lagerstroemia indica*) (source: Professor Tsuneo Ogata) and (**d**) the roots of three Pseudoacacia (*Robinia pseudoacacia*) trees.

**Figure 3:**
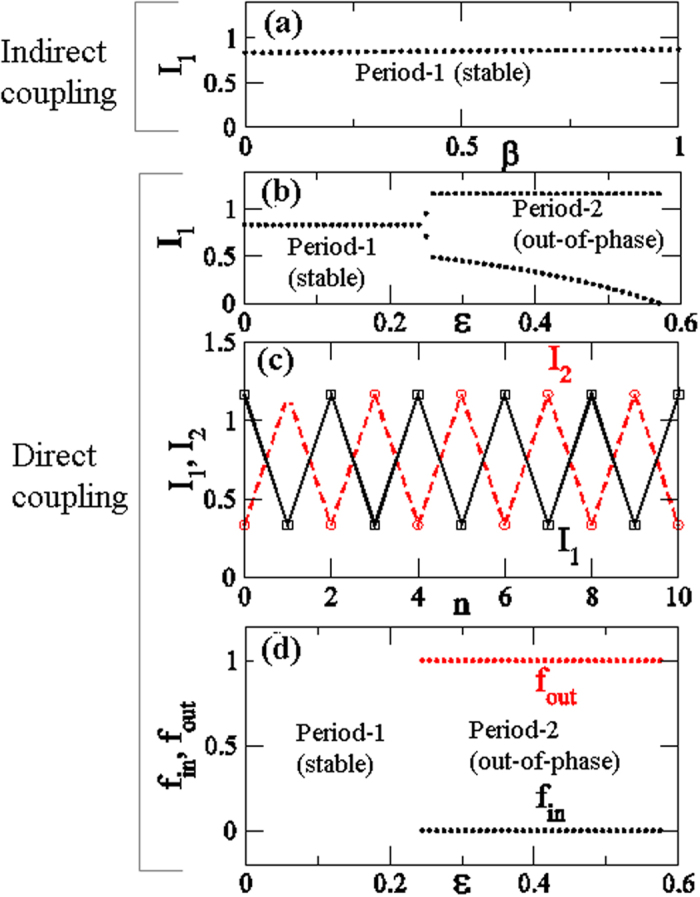
Coupling at Peridod-1 regime: R = 0.5. (**a**) The bifurcation diagram, *I*_1_, with the function of indirect (pollen) coupling strength *β*. For direct coupling: (**b**) the bifurcation, *I*_1_, as a function of coupling strength *ε*. (**c**) the trajectories of individual plants with time at coupling strength, *ε* = 0.4 and (**d**) the normalized fraction initial conditions with the function of coupling strength *ε*.

In the previous subsections we considered the effects of direct and indirect coupling separately. Now we consider the simultaneous presence of both these interactions in two coupled plants (Fig. 2(a)) for *R* = 0.5 (Period-1 regime:A1) and 1.2 (Multi-band chaotic regime, A2). The results are shown in Fig. 6 (a) and (b) at *R* = 0.5 and 1.2, respectively, in the plane *β−ε*. The contour in Fig. 6(a) illustrates the critical line (between red and black) where period-1 bifurcates into period-2. The line corresponds to the largest Lyapunov exponents (LLE) at zero^45^. This shows that in region P2 the behaviors of both the plants are out-of-phase (f_out_ > 0.8, similar to Fig. 3(b)). Figure 6(b) illustrates the contours map of *f*_out_ from 0.1 to 0.8. This shows that out-of-phase behavior is enhanced as direct coupling strength, *ε*, increases. Figure 6(a) and (b) confirm that out-of-phase behavior can exist in the presence of both types of interactions and that it increases with increasing *ε*. This implies that direct coupling induces out-of-phase behavior even under indirect interactions.

### Effect of mixed interactions in extended systems

Indirect interactions are a global phenomenon, affecting all elements in the systems in the same way. For example, in a given orchard, an external agent such as airborne pollen will affect all flowering plants equally (Eq.(4)). Figure 2(b) represents this example for three coupled plants, where pollen interactions (dashed line, *β* ≠ 0) exist between all the plants. In contrast, direct coupling is a local factor, and its interactions depend on the plants selected. Practically, it is almost impossible to couple all the plants in a system by grafting them together. To visualize the effects of direct coupling (in a few plants) and indirect coupling (in all available plants) in an orchard with many plants, we present a model for three representative, coupled plants, as in Fig. 2(b). Here, direct coupling exists only between two plants, while indirect coupling is present for all the plants. The dynamical model for this system becomes


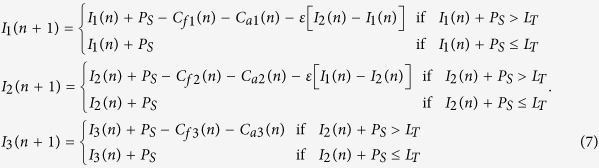



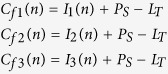



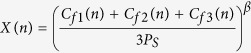



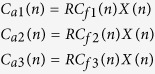


The results are shown in Fig. 7 (a) and (b) for *R* = 0.5 and *R* = 1.2, respectively. The different regions are marked as those in Fig. 6 for the first and the second plant. The results clearly show that direct coupling induces iut-of-phase synchronization as well as in Fig. 6.

**Figure 4:**
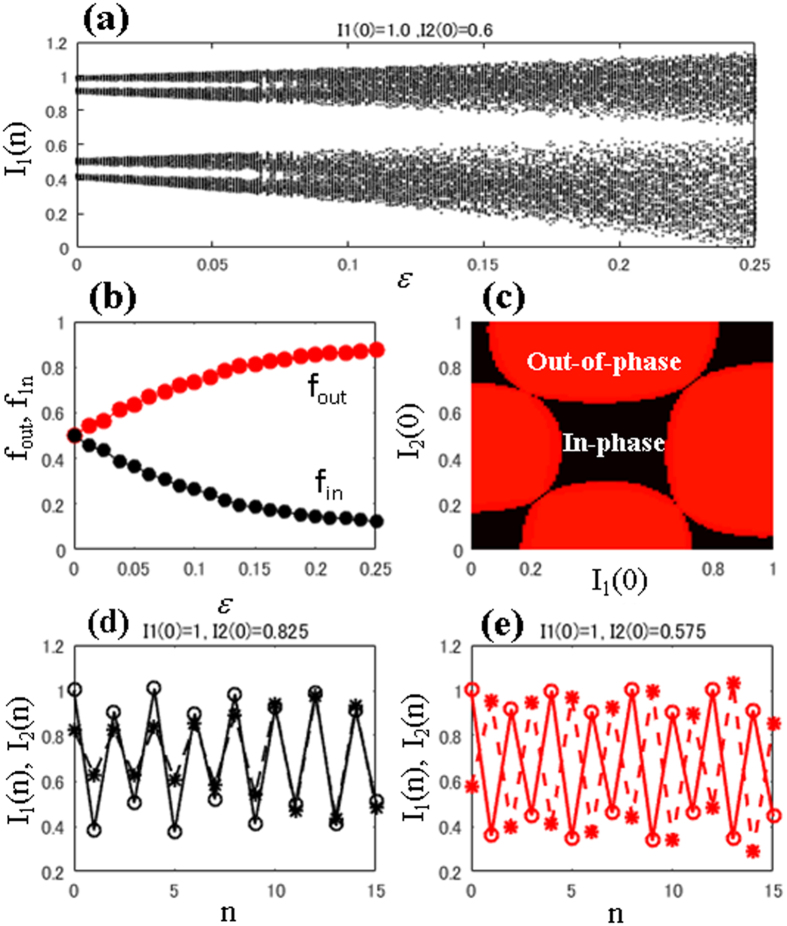
Direct coupling at multi-band chaotic regime, A2: *R* = 1.2. (**a**) The bifurcation diagram, *I*_1_, and (**b**) the fraction of the initial conditions corresponding to the in-phase (*f*_in_ -line) and out-of-phase (*f*_out_ -line) states as a function of *ε*. (**c**) The basins for the initial conditions for the in-phase and out-of-phase states are shown in black and red regions, respectively. The trajectories, *I*_1_ and *I*_2_, for coexisting (**d**) in-phase and (**e**) out-of-phase attractors at *ε* = 0.1 are shown.

Comparisons of the figures for two (Fig. 6) and three oscillators (Fig. 7) confirm that the out-of-phase behavior between plants can be dominant and persistent in significant range of the *β -ε* space. This also indicates that, in a given orchard, direct coupling should be able to induce out-of-phase behavior between directly coupled plants by setting appropriate initial conditions of two coupled trees to be out-of-phase. Note that the calculation in Figs 6 and 7 were carried out to a maximum of *ε* = 1, although the ecologically meaningful range may only reach the lower values seen in Figs 3 and 4.

### Experimental feasibility

In this section, we discuss the possibility of verifying the theoretical data obtained from modeling through field experiments and the potential limitations on such studies. Figure 3(d) shows that direct coupling induces out-of-phase behavior even in the period-1 regime (A1 region). Similarly Fig. 4(b) also suggests that the fraction of out-of-phase behavior is dominant for chaotic coupled behaviors. This out-of-phase dominance also persists in the presence of both types of interactions, as shown in Figs 6 and 7 for two and three coupled plants, respectively.

As these results show a higher likelihood of out-of-phase behavior occurring in the presence of both indirect interaction types, we may be able to use this property to control yearly fruit production. Let us suppose that we have *N* plants in a given orchard. If we are able to directly couple only *N*/2 pairs of plants (a single plant is directly connected to only one other plant), then we can control the total production of the orchard. That is, half of the plants will be in an “on-year” while the remaining half will be in an “off-year” owing to the dominance of out-of-phase dynamics. Therefore, the total production of the orchard across years will remain constant. Note that this strategy does not change the underlying action of alternate bearing, but it facilitates uniform production (shifting the fruit bearing timing of paired plants) by allowing only half of the plants to undergo fruit bearing in a given year.

Another very important observation can be drawn from Fig. 3 as follows. Suppose we wish to have constant production in just two plants. Let us achieve constant output in the uncoupled plants (as in regime A1 in Fig. 1) by proper thinning method^46^. If we are able to directly couple these plants, then we can expect out-of-phase production only for these plants, as shown in Fig. 3(b), at higher direct coupling strengths. This means that we can achieve constant output each year. This will result in a situation (as in Fig. 3(b)) in which we only obtain out-of-phase behavior at higher coupling strengths even in Period-1 regime(A1:R < 1). It is a remarkable advantage as a fruit production method for alternate bearing tree crops.

**Figure 5:**
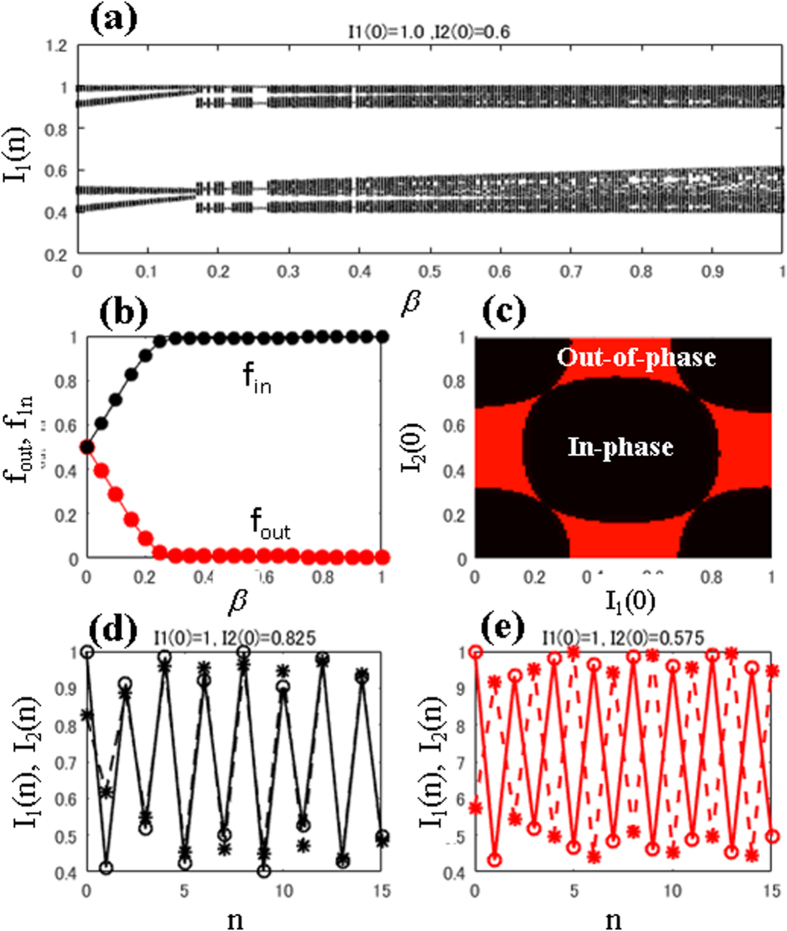
Indirect coupling at Peridod-2 regime: R = 1.2. (**a**) The bifurcation diagram, *I*_1_, and (**b**) the fraction of the initial conditions corresponding to the in-phase (*f*_in_ -line) and out-of-phase (*f*_out_ -line) states as a function of *β*. (**c**) The basins for the initial conditions for the in-phase and out-of-phase states are shown in black and red regions, respectively. The trajectories, *I*_1_ and *I*_2_, for coexisting (**d**) in-phase and (**e**) out-of-phase attractors at *β* = 0.1 are shown.

**Figure 6:**
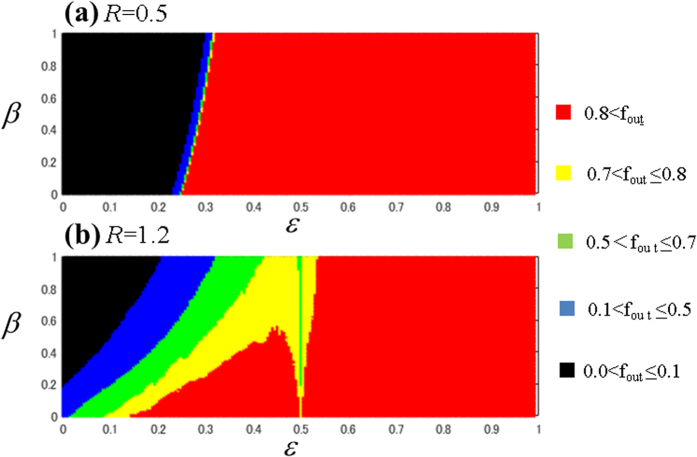
Two plants coupling: Schematic phase diagrams in parameters space, *β -ε*. (**a**) At *R* = 0.5 and (**b**) *R* = 1.2. P1 and P2 in (**a**) correspond to period-1 and 2 respectively. 5 Regions are demarcated after considering the contour on fraction of initial conditions leading to out-of-phase synchronized motions at 0.1 to 0.80.

**Figure 7:**
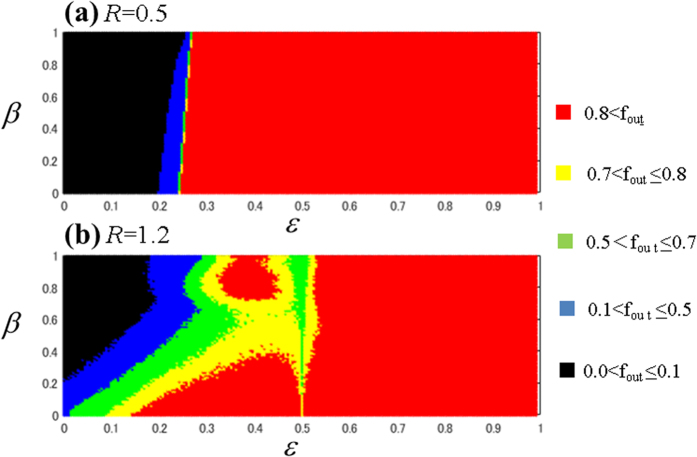
Three plants coupling: Schematic phase diagrams in parameters space, *β -ε*. (**a**) at *R* = 0.5 and (**b**) *R* = 1.2 for coupled three plants, model Fig. 2(b), in the presence of mixed interactions, Eq. (7). 5 Regions are demarcated after considering the contour on fraction of initial conditions leading to out-of-phase synchronized motions at 0.1 to 0.80.

Therefore, we suggest that this direct coupling method can be used to achieve constant total production of an orchard. One limitation facing experimental validation of this theory is the presence of coexisting in- and out-of-phase behaviors depending on their initial conditions, as shown in Figs 4(c) and 5(c). Because plants are affected by external factors, there is the possibility of either starting from the basin of in-phase behavior or switching between in- and out-of-phase behaviors. Importantly, as we see in Figs 4(c) and 5(c), the basin boundaries are smooth (i.e., the boundaries are not as complex as riddled^47^,^48^,^49^ or fractal ones) therefore known controller techniques^50^ and we can easily set the initial conditions for the out-of-phase state. If an appropriate and feasible technique for basin switching/control is designed, then direct coupling could be very useful. Thus, we propose here that direct coupling may be useful for future stable fruit production. We hope the theoretical results presented will stimulate initiatives to develop future applications for controlling constant fruit production.

### Summary

Considering the potential importance of ensuring constant fruit production in alternate-bearing plants, here we propose a theoretical concept based on nonlinear dynamics for controlling fruits production. We utilized the existing RBM, which captures many of the alternate-bearing plant phenomena. We coupled plants directly by grafting, which enhanced out-of-phase among plants. These directly coupled plants showed dominant out-of-phase synchronization, even in the presence of indirect interactions (e.g., pollen), suggesting the possibility of controlling fruit production. We show that once plants are directly coupled, it is possible to control plant behavior without further manual interference. That is, the proposed technique is automatic and self-sustaining. In this work, we offer the models for two and three coupled plants in the presence/absence of direct and indirect interactions. These models had similar results, suggesting that the conclusions presented here would likely apply to orchards with many plants. We have utilized one well-known model that captures many properties of the alternate-bearing phenomenon. However other variants of this model, such as those described in other studies^15^,^46^,^47^, could be considered for generalizing the presented results. Verifying the theoretical results presented here through field experiments is possible, but will take many years, perhaps decades. Above we have discussed the feasibility and potential limitations of field studies. We believe this initial proposal for controlling production in alternate-bearing plants could serve as a basis for further development of this concept.

## Additional Information

**How to cite this article**: Prasad, A. *et al*. Direct coupling: a possible strategy to control fruit production in alternate bearing. *Sci. Rep.* **7**, 39890; doi: 10.1038/srep39890 (2017).

**Publisher's note:** Springer Nature remains neutral with regard to jurisdictional claims in published maps and institutional affiliations.
