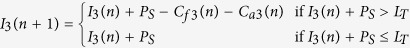# Corrigendum: Direct coupling: a possible strategy to control fruit production in alternate bearing

**DOI:** 10.1038/srep45251

**Published:** 2017-03-30

**Authors:** Awadhesh Prasad, Kenshi Sakai, Yoshinobu Hoshino

Scientific Reports 7: Article number: 3989010.1038/srep39890; published online: 01 04 2017; updated: 03 30 2017

This Article contains a typographical error in the Results and Discussion section. In Equation (7),


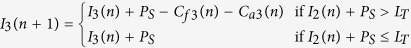


should read: